# A protease-resistant *Escherichia coli* asparaginase with outstanding stability and enhanced anti-leukaemic activity *in vitro*

**DOI:** 10.1038/s41598-017-15075-4

**Published:** 2017-11-03

**Authors:** Maristella Maggi, Steven D. Mittelman, Jean Hugues Parmentier, Giorgio Colombo, Massimiliano Meli, Jeannette Marie Whitmire, D. Scott Merrell, Julian Whitelegge, Claudia Scotti

**Affiliations:** 1 Department of Molecular Medicine, Unit of Immunology and General Pathology, University of Pavia, Pavia, Italy; 2 Center for Endocrinology, Diabetes & Metabolism, Children’s Hospital Los Angeles, Los Angeles, CA USA; 3 Biomolecular Simulations & Computational Chemistry Group, Istituto di Chimica del Riconoscimento Molecolare, CNR, Milan, Italy; 4 Department of Microbiology and Immunology, Uniformed Services University of the Health Sciences, Bethesda, MD USA; 5 Julian Whitelegge, The Pasarow Mass Spectrometry Laboratory, The NPI-Semel Institute & Jonsson Comprehensive Cancer Center, David Geffen School of Medicine, UCLA, USA

## Abstract

L-Asparaginases (ASNases) have been used as first line drugs for paediatric Acute Lymphoblastic Leukaemia (ALL) treatment for more than 40 years. Both the *Escherichia coli* (EcAII) and *Erwinia chrysanthemi* (ErAII) type II ASNases currently used in the clinics are characterized by high *in vivo* instability, short half-life and the requirement of several administrations to obtain a pharmacologically active concentration. Moreover, they are sensitive to proteases (cathepsin B and asparagine endopeptidase) that are over-expressed by resistant leukaemia lymphoblasts, thereby impairing drug activity and pharmacokinetics. Herein, we present the biochemical, structural and *in vitro* antiproliferative characterization of a new EcAII variant, N24S. The mutant shows completely preserved asparaginase and glutaminase activities, long-term storage stability, improved thermal parameters, and outstanding resistance to proteases derived from leukaemia cells. Structural analysis demonstrates a modification in the hydrogen bond network related to residue 24, while Normal Mode-based geometric Simulation and Molecular Dynamics predict a general rigidification of the monomer as compared to wild-type. These improved features render N24S a potential alternative treatment to reduce the number of drug administrations *in vivo* and to successfully address one of the major current challenges of ALL treatment: spontaneous, protease-dependent and immunological inactivation of ASNase.

## Introduction

L-Asparaginases (ASNases) were described as antiproliferative agents in the 1950s^1^. Their therapeutic mechanisms rely on the removal of L-asparagine and L-glutamine from blood, taking advantage of the fact that most leukaemia cells are unable to *de novo* synthesize sufficient quantities of these amino acids^2^. ASNases are first line drugs for paediatric Acute Lymphoblastic Leukaemia (ALL). Presently, *Escherichia coli* (EcAII) type II ASNase, either in native or PEGylated form, is typically used as a first choice drug, while the *Erwinia chrysanthemi* (ErAII) enzyme is used only in cases of immune reactions to *E*. *coli-*derived ASNases. Unfortunately, ASNase-based therapy has some limitations: (i) pancreas, liver and immune system impairment, (ii) high immunogenicity, (iii) low efficacy and higher toxicity in adult patients, and (iv) low stability *in vivo* ^3,4^. In fact, both native proteins are characterized by low *in vivo* stability, resulting in short half-life and need for several administrations. Moreover, protein instability contributes to epitope unmasking, with an increased likelihood of antibody production and subsequent immune reactions^5^. Two lysosomal proteases, asparaginase endopeptidase (AEP) and cathepsin B (CTSB), further inactivate EcAII by hydrolysis, contributing to therapy failure^6^. AEP and CTSB are expressed by Philadelphia positive (Ph+) and iAMP21 leukaemia cells, two very aggressive and poorly responsive forms of leukemia^7–9^, while microenvironment cells such as macrophages can express CTSB and contribute to ASNase degradation^10^. While PEGylation improves ASNase stability and pharmacokinetic/pharmacodynamics profiles, immunogenicity and CTSB hydrolysis still limit its use for the treatment of ASNase-inactivating tumors^6^.

Biochemically, bacterial type II ASNases are amidohydrolases (EC 3.5.1.1) that catalyze L-asparagine (L-Asn) hydrolysis to L-aspartic acid (L-Asp) and ammonia. With reduced efficiency, ASNases are also capable of deaminating L-glutamine (L-Gln), releasing L-glutamic acid (L-Glu) and ammonia. From the structural point of view, ASNases are homotetramers of roughly 130–140 kDa. The N-domain contains the active site flexible loop (residues 3–27), a peculiar structural element that acts as a lid closing on the active site upon substrate binding and that carries two essential catalytic residues (a Thr and a Tyr).

Site-directed mutagenesis is routinely used to build EcAII variants with improved features, mainly aimed at modulating the enzyme substrate specificity and immunogenicity, and at improving its stability^11–14^. The active site flexible loop is a critical region for all these aspects, and, particularly, residue Asn24, even though not directly involved in catalysis, plays a crucial role in stabilizing the enzyme active site, interacting mainly with the catalytic T12 residue^15^. Moreover, Asn24 has been identified as primary cleavage site for both AEP and CTSB^16^. We designed an N24S mutation based on the assumption that this modification should lead to relevant changes in these interactions. Herein, we present the biochemical, structural and *in vitro* antiproliferative characterization of N24S, showing preserved activities, very much improved thermal and proteolytic resistance, and improved long-term cytotoxicity *in vitro*. These enhanced features provide a molecule potentially suitable to improve efficacy, particularly in drug-resistant patients and/or relapsed ALL.

## Results

### N24S has preserved specific activities and kinetic parameters

Kinetic parameters (Table 1) of the recombinant wild-type (WT) and N24S were very similar to each other, and to the deposited values (BRENDA enzyme database^17^). Both enzymes had a hyperbolic response of activity *versus* both L-Asn and L-Gln increasing concentrations, and a stronger preference for L-Asn over L-Gln as a substrate.

**Table 1:** Kinetic parameters of EcAII variants.

Variant	Specific Activity (U/mg)		K_m_ (mM)		k_cat_ (sec^−1^)		Efficiency (k_cat_/K_m)_	
	L-Asn	L-Gln	L-Asn	L-Gln	L-Asn	L-Gln	L-Asn	L-Gln
Wild-type	105.20 ± 8.14	0.89 ± 0.22	0.03 ± 0.01	3.95 ± 0.47	59.77 ± 4.63	0.51 ± 0.13	1975	0.13
N24S	103.50 ± 10.23	0.93 ± 0.20	0.03 ± 0.01	4.14 ± 0.33	58.81 ± 5.81	0.53 ± 0.012	1782	0.13
Wild-type*	—	—	0.04 ± 0.30	3.50 ± 0.50	49 ± 0.30	0.33 ± 0.20	1600	0.09

Experiments are average ± SD of at least 2 independent experiments performed in triplicate.

*Recombinant wild type EcAII described by^14^.

### N24S shows a high thermal resistance

**Figure 1:**
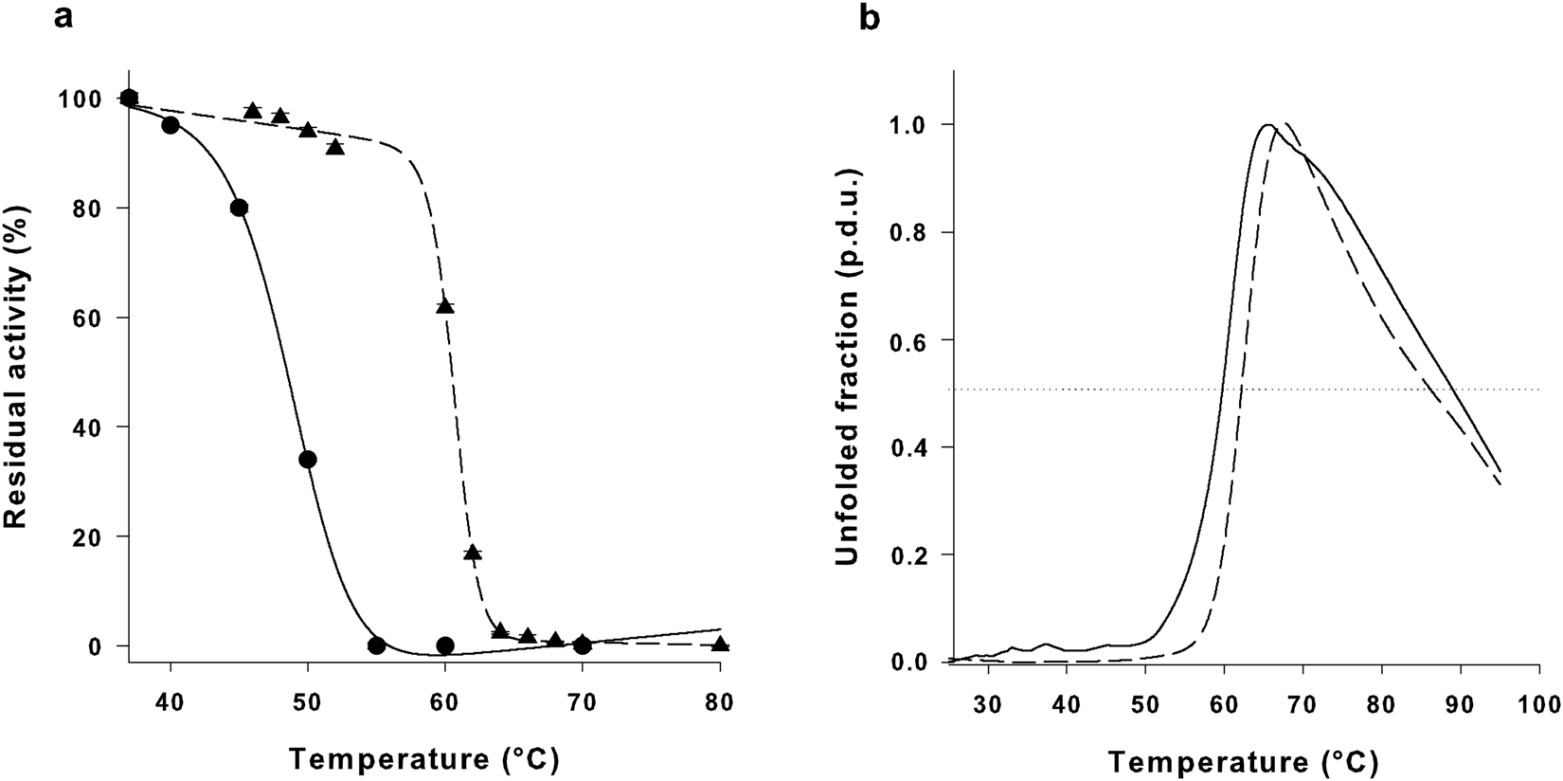
Thermal stability assays. (**a**) EcAII WT (circle) and N24S (triangle) thermal inactivation plot. Results are reported as residual activity percentage of heat-treated *vs*. untreated sample. Samples were incubated at 37 °C for 10 min and the residual activity was measured in the presence of 10 mM L-Asn. (**b**) Heat-induced melting curves of WT (continuous line) and N24S EcAII (dashed line). The horizontal dotted line is the reference line corresponding to an unfolded fraction value equal to 0.5. Melting curves were obtained by thermal shift analysis using CYPRO orange as a fluorescent dye. Unfolded fraction values are reported as procedure defined units (p.d.u.).

Because *in vivo* ASNase activity is related to thermal stability^18,19^, we next evaluated the melting temperature (T_50_) of our mutant enzyme *vs*. WT. The T_50_ of N24S was significantly higher than WT EcAII (59 ± 2.2 *vs*. 49 ± 1.3 °C, p = 0.002, Fig. 1a). N24S was also more resistant to heat-induced unfolding, as demonstrated by a rightward shift in thermal shift analysis (Fig. 1b). N24S T_0.5_ (the temperature at which half of the protein was in an unfolded state) was also higher than the WT one (62.04 ± 0.10 °C *vs*. 59.63 ± 0.62 °C, p < 0.001). Moreover, N24S had a slower unfolding kinetics than WT (5.3 *vs*. 7.5%/°C), and a higher initial unfolding temperature (57 *vs*. 51 °C).

### N24S shows long-term stability upon storage

**Figure 2:**
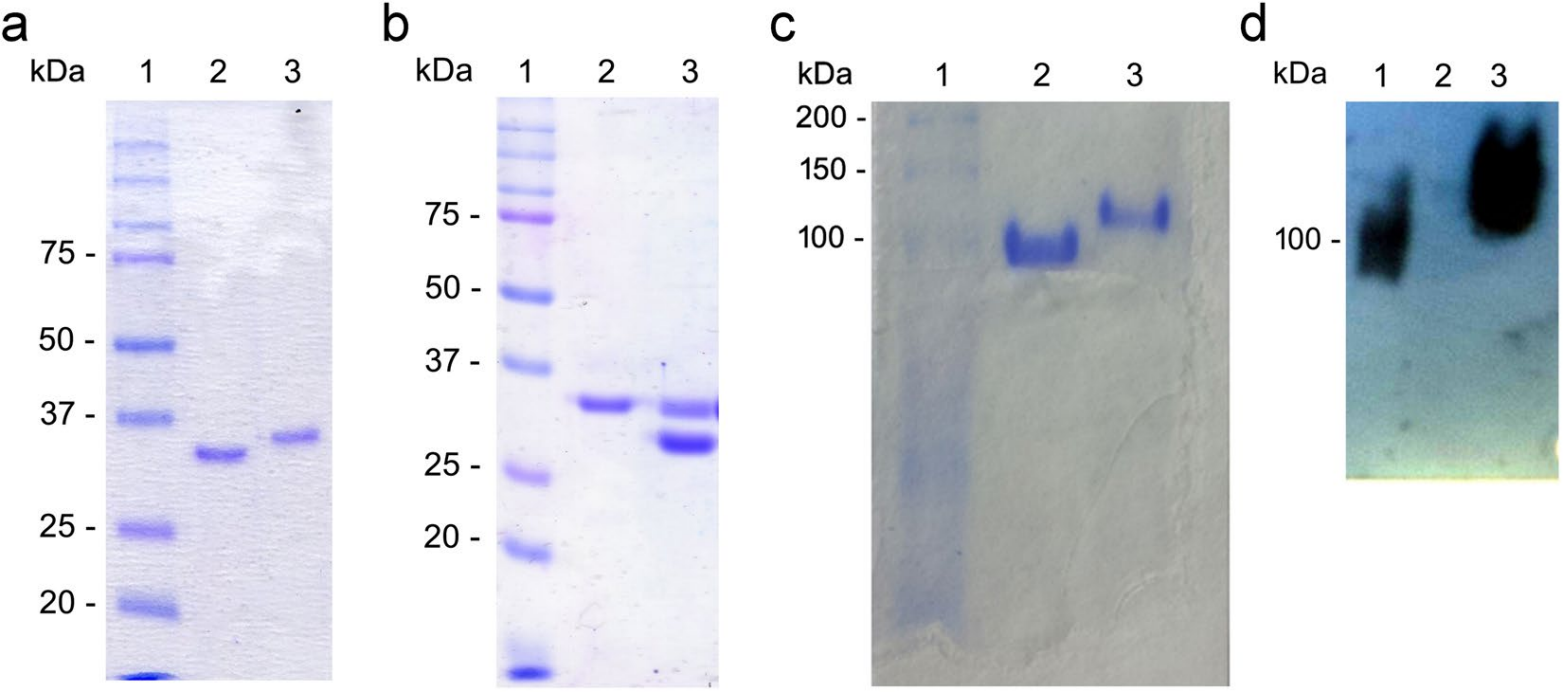
EcAII degradation pattern upon storage. (**a**) SDS-PAGE of EcAII after storage at +4 °C in Na-phosphate. Lane 1: molecular weight standards; lane 2: degraded protein (83-days old); lane 3: freshly purified protein (1-day old). (**b**) SDS-PAGE of EcAII and N24S after 1 yr storage at +4 °C and in PBS buffer. Lane 1: molecular weight standards; lane 2: N24S; lane 3: wild-type. (**c**) Native-PAGE of fully degraded (lane 2) and freshly purified EcAII (lane 3). (**d**) Western blot analysis of fully degraded (lane 2) and freshly purified EcAII (lane 3) after native-PAGE. Full-length blot is presented in Supplementary Figure 3.

Protein degradation was monitored by SDS-PAGE analysis and Coomassie staining. Freshly prepared EcAII run in reducing conditions typically showed a single 33 kDa band corresponding to the full-length, undegraded monomer (Fig. 2a, lane 3). Upon storage in the presence of water solution, progressive degradation and activities reduction occurred, while a 30 kDa band became predominant over time: in Fig. 2a, lane 2, a preparation preserved in Na-phosphate buffer at +4 °C for 83 days is shown. This behaviour was very consistent among different batches and not due to contaminating proteases^20^ (data not shown). In fact, a qualitative test for proteases detection both on freshly purified and degraded protein solutions was performed. The test was based on the use of casein as a substrate and Folin-Ciocalteu as a detection dye^21^. In all cases, protein solutions were negative for proteases. Compared to the typical 7–10 days stability of WT in Na- phosphate at 4 °C, N24S was active for more than 9 weeks. It did not show any sign of degradation when preserved in PBS at +4 °C even after one year (Fig. 2b, lane 2), in contrast to the WT (Fig. 2b, lane 3). Both proteins in lyophilized form showed full stability after 1 year at −20 °C (data not shown). Though N24S showed long-term stability upon storage, the underlying mechanism needs to be clarified.

An aliquot of fully degraded EcAII WT was analyzed by native-PAGE in order to determine if, despite its progressive loss of activity, the tetrameric complex was maintained upon degradation. As shown in Fig. 2c, freshly purified EcAII ran as a single band with a molecular weight (MW) comprised between 100 and 150 kDa (lane 3), while the fully degraded and inactive protein (lane 2) ran as a single species with a slightly lower MW (ca. 100 kDa). The absence of other bands of different MWs suggests preservation of the tetrameric complex upon degradation. The fully degraded protein (Fig. 2d, lane 2) was not reactive towards the anti-HisTag antibody directed to the EcAII N-terminus 6xHisTag, suggesting removal of the N-terminal portion of the protein.

A solution containing a partially degraded EcAII WT was then analyzed by N-terminal sequencing to determine cleavage points. The obtained results showed that, apart from the full-length protein, a mixture of protein forms with an N-terminus starting within amino acids 25–30 was detected, indicating a complete removal of the flexible loop residues (3–27).

### N24S has a higher long-term cytotoxicity than the wild-type

**Figure 3:**
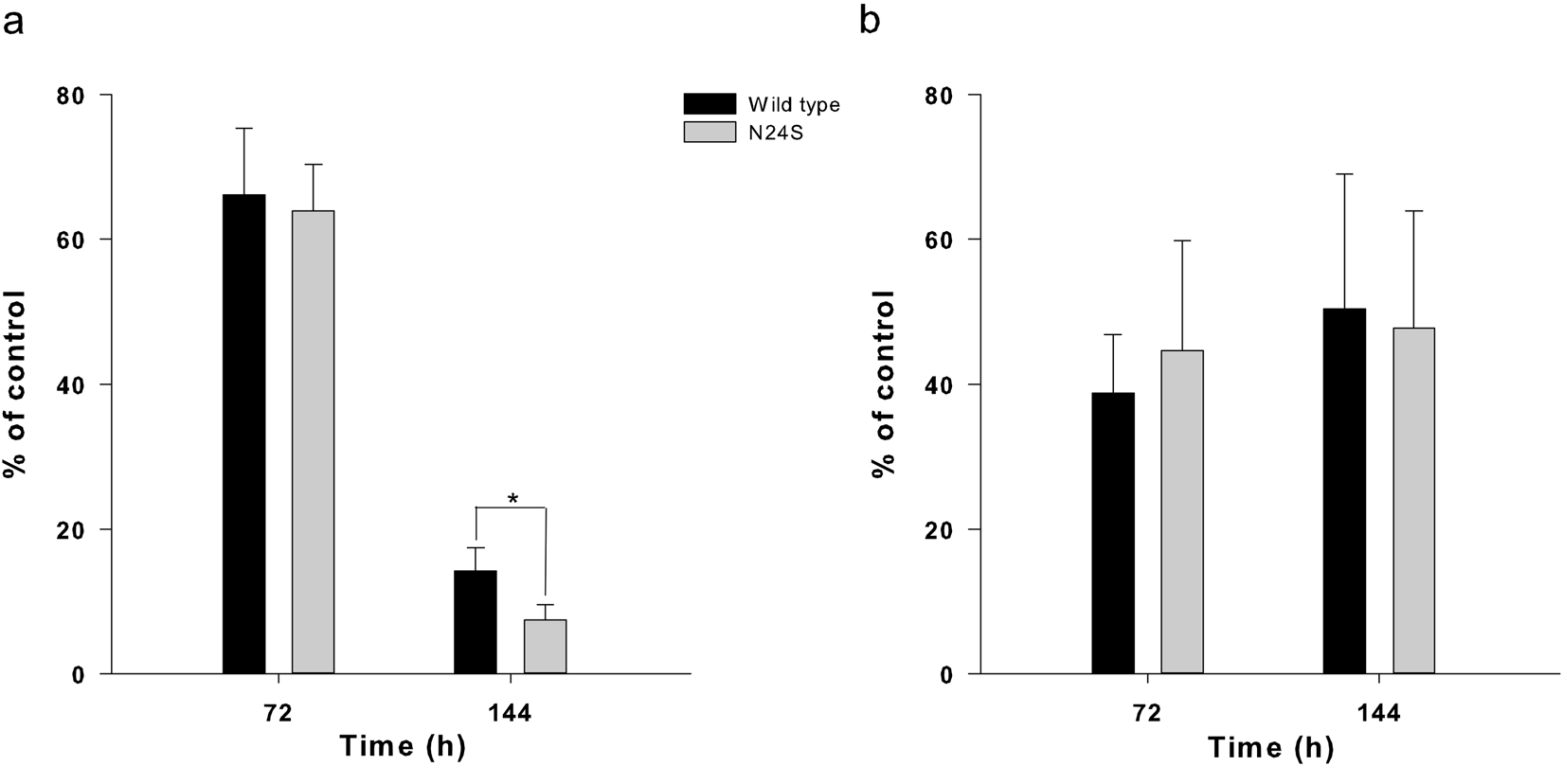
Effect of WT EcAII and N24S on SD1 (**a**) and REH (**b**) cells. Data are the average ± SD of at least 3 independent experiments performed in triplicate. Statistical significance (p < 0.05) was evaluated by one-way ANOVA and Tukey test. Untreated cells (Ctrl) were set as 100%.

In order to compare the *in vitro* anti-proliferative effect of EcAII WT and N24S to the commercial enzyme Elspar (Medac), cytotoxicity assays were performed against several human ALL cell lines (MOLT-4, REH, RS4;11 and SD1). EcAII WT and N24S showed a cytotoxic effect comparable to the commercial Elspar protein on all cell lines but SD1, where, unexpectedly, they had a nearly 4.30-fold higher cytotoxic effect at 3.0 U/ml (Table 2). Regarding long-term cytotoxicity, WT and N24S had the same effect on both SD1 and REH cells at 72 h. At 144 h, however, N24S had a 47.5% higher cytotoxicity on SD1 cells than the WT (p = 0.000), while both ASNases had the same cytotoxicity on REH cells (Fig. 3a,b).

**Table 2:** Cytotoxicity of EcAII wild-type, N24S and Elspar.

Cell line	U/ml	Viability (%)		
		EcAII	N24S	Elspar
MOLT-4	0.005	29.92 ± 3.47	28.21 ± 1.35	28.85 ± 0.77
REH	3.0	55.21 ± 2.80	61.80 ± 6.52	58.8 ± 11.55
RS4;11	0.005	29.30 ± 5.57	24.0.3 ± 3.56	25.15 ± 1.39
SD1	3.0	19.88 ± 6.86	21.72 ± 4.96	84.51 ± 8.60

Experiments are average ± SD of at least 2 independent experiments performed in triplicate.

### N24S is insensitive to SD1 cell proteases

**Figure 4:**
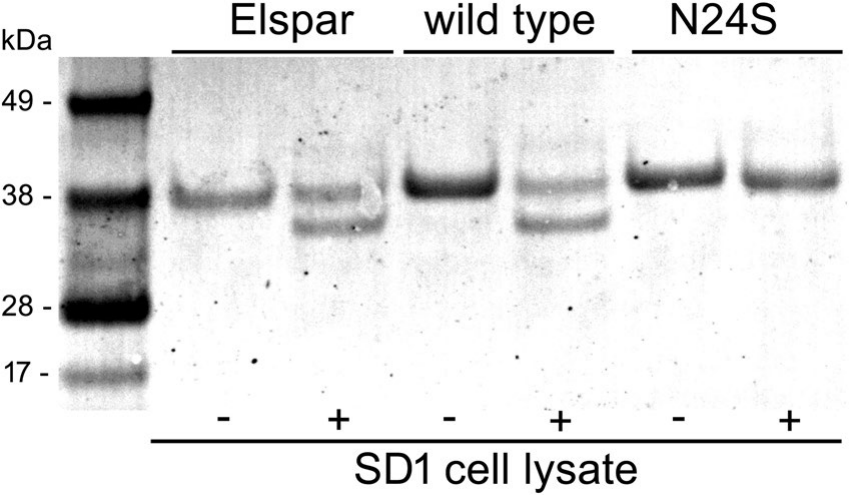
Elspar, EcAII WT and N24S degradation by SD1 cell lysate (Ponceau Red staining). +/− indicates the presence or absence of cell lysate.

EcAII WT, N24S and Elspar were incubated in the presence of SD1 cell lysate at 37 °C o.n. After incubation, samples were separated by SDS-PAGE and blotted onto a nitrocellulose membrane. After Ponceau Red staining, a clear degradation band was evident in the WT and Elspar samples and absent in the N24S sample (Fig. 4). The experiment was repeated 4 times with similar results. Protein masses were measured by low-resolution ESI-MS after treatment with cell extracts. The measured mass of ASNase before cleavage (35548 Da) was in good agreement with that calculated for the His-tagged monomer (calculated average mass 35546 Da). The measured mass of ASNase after cleavage (31963 Da) was consistent with an N-terminal cleavage at G28 (calculated average mass 31962 Da) within experimental error. A less abundant species at 32116 Da was consistent with an N-terminal cleavage at T26 (calculated average mass 32118 Da). The observed 2 amino acid steps from N24 might arise from N-exo-dipeptidase trimming after N-terminal processing.

### N24S shows a global structural organization similar to EcAII wild-type

Single prismatic crystals of N24S were obtained in 100 mM HEPES, pH 7.5, 5% w/v PEG 8000, 4% v/v ethylene glycol, at 21 °C after 4 days. The crystal described in the present paper had an approximate size of 300 × 35 × 30 µm and diffracted up to 1.60 Å resolution. Crystallographic data and statistics of the best dataset are summarized in Table 3. The crystal asymmetric unit (ASU) contained 2 distinct intimate dimers (namely, A/C and B/D), which rebuilt the full biological tetramer (ACBD) by crystallographic symmetry (for clarity, in the following text Ser24(A) indicates Ser24 of monomer A). Clear electron density (ED) corresponding to the L-Asp product was present in all 4 active sites of the ASU. Two of the 4 monomers (i.e., C and B) also presented clear electron density for residues belonging to the active site flexible loop (residues 3–27), while residues 16–27 were missing in monomers A and D. Overall, the monomers present in the ASU were similar (r.m.s.d. average of the main chain: 0.19 ± 0.05 Å, Supplementary Table 1). The overall structure of the N24S tetramer, the orientation and organization of critical catalytic residues, and of the L-aspartate product are highly similar to the WT one (r.m.s.d. 0.34 ± 0.01 Å, supplemental Tables 4–5 and supplemental Fig. 1).

**Table 3:** Crystallographic data and statistics for N24S. High resolution shell is shown in parenthesis.

	5MQ5
Data collection	
Space group	C 2
Cell dimensions	
*a*, *b*, *c* (Å)	152.0, 62.34, 142.27
α, β, γ (°)	90.0, 117.92, 90.0
Resolution (Å)	48.16–1.43 (1.43–1.50)
*R*_*merge*_	0.062 (1.24)
Mean I/σI	8.60 (0.60)
Completeness (%)	89.70 (57.90)
Redundancy	2.60 (2.0)
Refinement	
Resolution (Å)	12.97–1.60 (1.66–1.60)
No. of reflections	275508 (27697)
*R*_*work*_ /*R*_*free*_	0.80 (0.89)
No. atoms	
Protein	9600
Ligand	36
Water	1262
*B*-factors	
Protein	25.87
Ligand	21.83
Water	31.65
R.m.s. deviations	
Bond length (Å)	0.011
Bond angles (°)	1.0

**Table 4:** Persistent H-bonds.

Protein	Residue (Chain)	Residue (Chain)	Percentage of presence (%)
N24S + ASP	Asp18(A)	Ser24(A)	26.0
	Asp18(A)	Ser24(A)	21.4
	Lys22(A)	Ser24(A)	20.7
WT + ASP	Asp281(C)	Asn24(A)	29.1
N24S APO	Lys22(A)	Ser24(A)	20.8
	Asp280(B)	Ser24(A)	16.2
WT APO	Ala282(C)	Asn24(A)	24.8

The data are reported for 1 single monomer, but in the case of N24S the same pattern is seen across all monomers.

**Table 5:** Normal geometric-Mode Simulations.

Loop motions	Residues with relevant r.m.s.f. Δ	Residues with Δ r.m.s.f. >0.5 Å	Residues with Δ r.m.s.f. <0.5 Å
Lowest ROG	139 of 326	14 of 139	125 of 139
Largest ROG	53 of 326	3 of 53	50 of 53
**Protein motions**	**Conformations with relevant r**.**m**.**s**.**d**. **Δ**	**Conformations with Δ r**.**m**.**s**.**d**.** > 0**.**5 Å**	**Conformations with Δ r**.**m**.**s**.**d**.** < 0**.**5 Å**
Lowest ROG	520 of 1497	0 of 520	520 of 520
Largest ROG	787 of 1467	0 of 787	787 of 787

N24S residues (loop motions) or conformations (protein motions) with higher (Δ r.m.s.f. or d. > 0.5 Å) or lower (Δ r.m.s.f. or d. < 0.5 Å) than the WT.

### Local structural changes indicate a reorganization of internal and inter-monomer interactions in N24S

**Figure 5:**
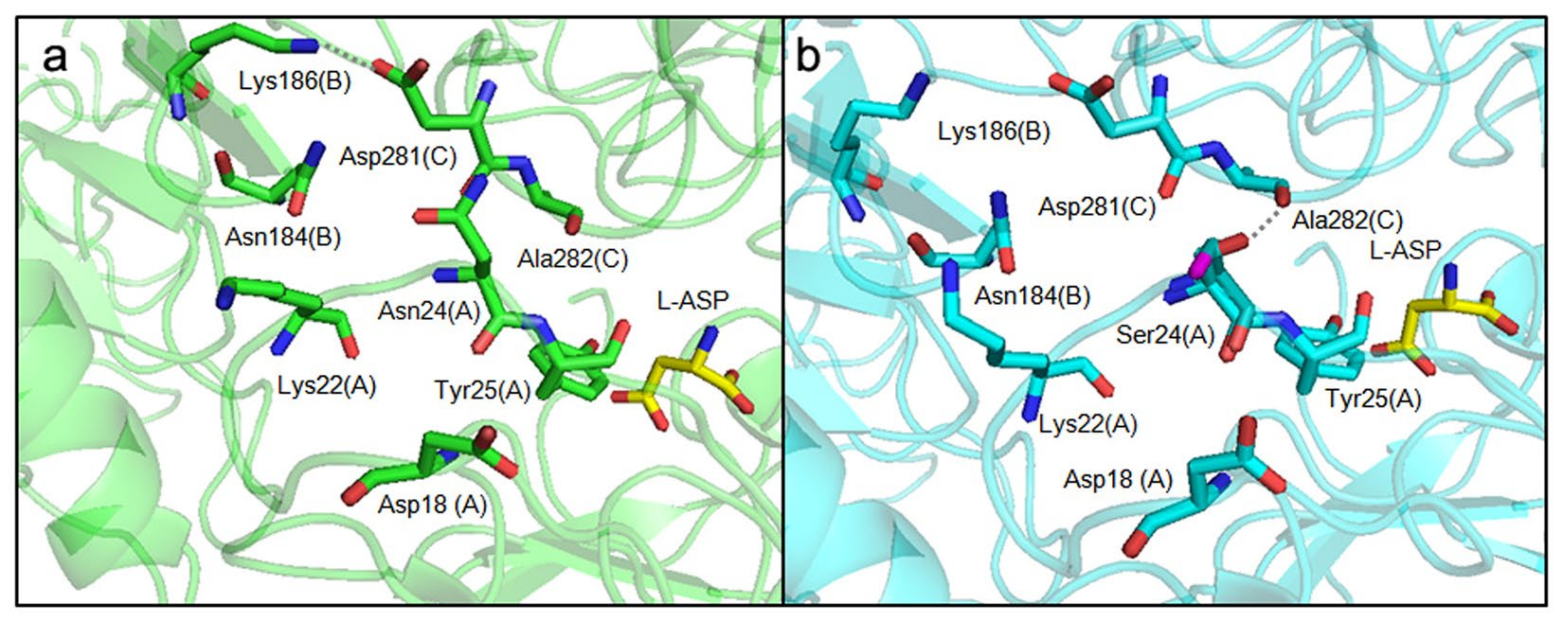
Comparison of H-bonds of Asn24 (EcAII WT, panel a) and of Ser24 (N24S, panel b). Only H-bonds predicted by PISA and different in the two structures are shown and are represented as dotted lines. Relevant residues are shown as sticks colored in green for the WT and in cyan for the N24S mutant. The two conformations of Ser24 Oγ in the N24S mutant are shown in purple (monomer B) or in red (monomer C). The L-Asp reaction product is shown for both structures and represented as sticks (yellow). For each residue the monomer to which it belongs is annotated as a capital letter in parentheses.

In the WT structures (e.g., 3ECA and 1NNS), the residue Asn24 side chain faces the near intimate monomer and acquires contacts with it through interactions with residue Asp281 (Fig. 5)^15^. Two different conformations of residue Ser24 are visible in the two monomers (B and C), where ED for the loop was clearly available (Fig. 5b). In the conformation present in monomer B, the Ser24 Oγ group is oriented towards the inner part of the flexible loop. In the conformation present in monomer C, the Ser24 Oγ group is oriented towards the near intimate monomer (chain A) and acquires contacts with the main chain O of residue Ala282. The shortening of the residue 24 side chain in the mutant protein causes the loss of contacts with the side chain of D281 in the intimate dimer, while the presence of the Ser OH group weakens the bond compared to the one formed by the Asn NH_2_ group. The bond formed by Asp281(C) Oδ2 and Lys186(B) NHζ (Fig. 5) is also lost in the mutant structure. Molecular dynamics simulations on wild type and on N24S L-Asp-bound proteins show the presence of a persistent H-bond between Asn24(A) and Asp281(C) that is lost in the mutant structure in favour of two new intra-loop persistent H-bonds between Ser24(A) and Asp18(A) and Lys22(A) (Table 4). Analysis of the two proteins in their apo form shows a similar behaviour consisting in the loss in the mutant of one inter-monomer persistent H-bond (Asp24(A) - Ala282(C)) and the formation of two new persistent H-bonds, one intra-monomer (Ser24(A) - Lys22(A)) and one inter-monomers (Ser24(A) - Asp281(C)).

### Normal Mode-based geometric Simulation and Molecular Dynamics show a general rigidification of N24S

A general tendency towards rigidification of N24S (that is, a lower r.m.s.f.) with respect to the WT was observed by Normal Mode-based geometric Simulation (Supplementary Figure 2a, Table 5). The highest rigidification was registered for the 166–168 loop with a Δ r.m.s.f. between 1.15 and 1.38 Å. Protein motions analysis, using the lowest radius of gyration (ROG) as conformation choice criteria, showed a relevant variation in 520 of the 1497 total conformations. Moreover, all the 520 variations had a lower r.m.s.d. (Δ comprised between −0.5 and −1.11 Å) for N24S than for the wild-type (Supplementary Figure 2b, Table 5). Analysis of protein motions, using the highest ROG as conformation choice criteria, showed that all the conformations had r.m.s.d. values lower for N24S (Δ range, −0.5/−1.19 Å) than for the wild-type (Supplementary Figure 2c, Table 5). Loop motion was evaluated for the more extended conformations (largest ROG, Supplementary Figure 2d). Only 3 residues had a r.m.s.f. higher for the mutant (i.e., residues 21 and 166–168, Δ range 0.7–0.8 Å), the other 50 residues, comprising residues 23–30, all had an r.m.s.f. lower for the mutant in a Δ range −0.5 and −1.7 Å (Table 5).

**Figure 6:**
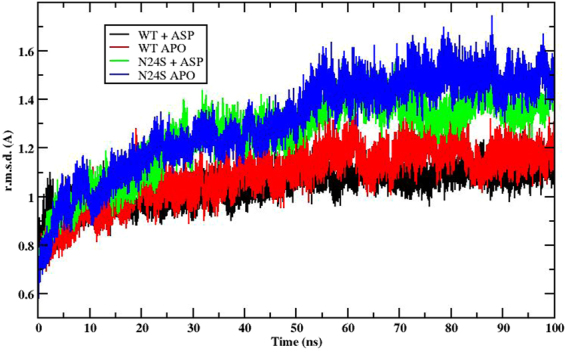
RMSD (Å) as a function of simulation time (ns). Both the wild-type and the N24S mutant show oscillations below 1.5 Å, indicating no major structural distortion.

Molecular Dynamics (MD) simulations confirmed and extended these results. The atom-positional r.m.s.d. from the crystal structures of the WT and N24S proteins in both the apo and Asp-bound forms are displayed in Fig. 6. All simulations show r.m.s.d. values lower than 1.5 Å, consistent with the fact that no major structural distortion is taking place in the crystals.

**Figure 7:**
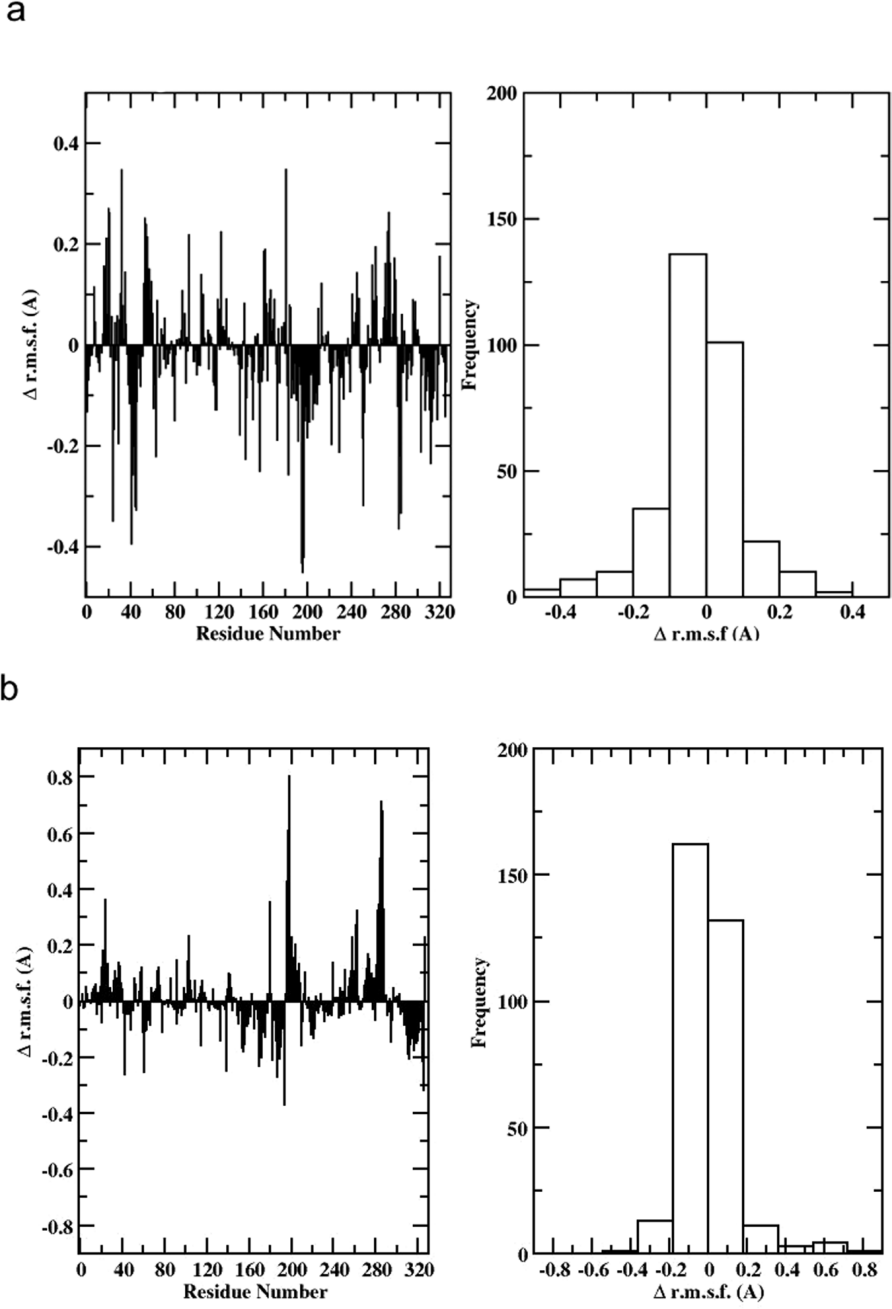
RMSF (Å) of N24S versus wild-type in (**a**) apo form and (**b**) ligand-bound. Negative values along the sequence indicate a rigidification in the mutant.

To investigate protein flexibility and its relationships to mutation-induced stabilization, we calculated the r.m.s.f. for residues around their equilibrium positions during the simulations. In this case, the r.m.s.f. value was calculated for the different residues in each monomer, and then the values for corresponding residues in the different monomers were averaged to provide an approximate measure of protein flexibility. Next, we calculated the difference r.m.s.f. between N24S and WT, by subtracting the averaged values of the corresponding residue positions. In Fig. 7a and b, negative values along the sequence indicate a rigidification in the mutant. The predominance of negative values shown in the histograms reporting the distribution of r.m.s.f.-differences indicate a general rigidification of the mutant, both in the apo and in the bound states. Structural rigidification can aptly reverberate in structural stabilization and resistance to unfolding or degradation.

**Figure 8:**
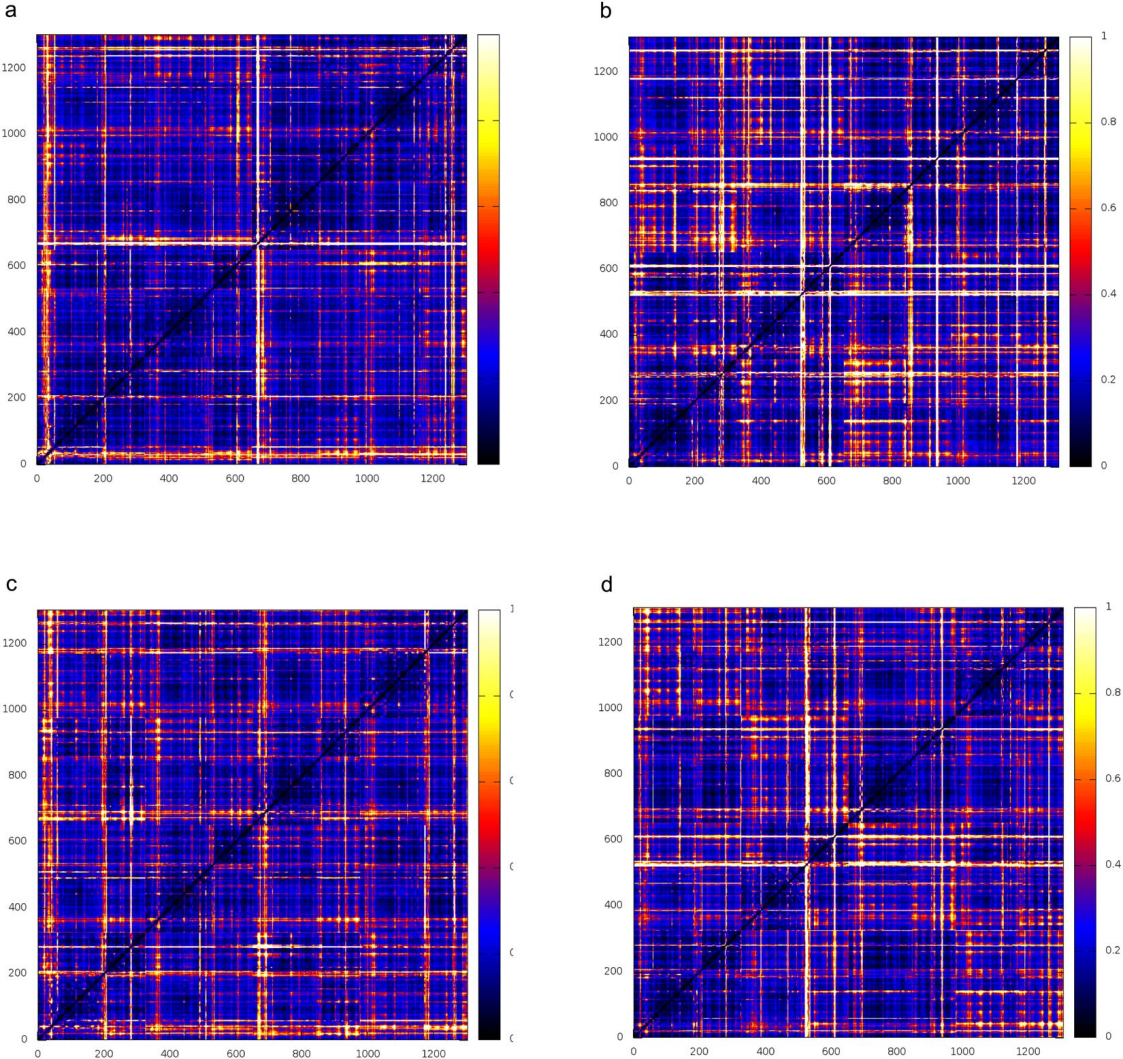
Fluctuations of pairwise amino acid distances on all possible residue pairs. (**a**) N24S apoprotein; (**b**) N24S bound to aspartate; (**c**) wild-type apoprotein; (**d**) wild-type bound to aspartate.

We then extended our analysis of mutation-induced dynamic rigidification to the global level, by calculating the fluctuations of pairwise amino acid distances on all possible residue pairs in the protein (Fig. 8)^22–24^. The same calculation was then carried out separately for each monomer. This calculation reports on the distribution of rigid vs. flexible substructures in the protein by evaluating the long-range coordination of residue pairs in the structure (the lower the distance fluctuations the higher the coordination between two residues). Groups of amino acids (even belonging to different domains) may respond to mutation by changing their long-distance coordination, coupled to a modulation of the inter domain rigid dynamics. All matrices exhibited a similar block character which reflects the presence of regions of small and large fluctuations of inter-residue distances corresponding to the various monomers. Nevertheless, N24S in the apo state, and to a lesser extent in the Asp-bound state, are characterized by a diffuse coordination (low fluctuations) pattern among residues that diffuses throughout the protein structure: extended coordination indicates an increased overall rigidity of the mutated tetramer. The observed global rigidification for the mutant can aptly be considered to oppose conformational changes that may guide the protein along unfolding pathways.

Finally, we analyzed the H-bonding patterns that characterize the active site in the two proteins. The most persistent H-bonds are reported in Table 4. It is clear from the data that the N24S mutation modifies the H-bonding distribution favoring the formation of intramonomer interactions, compared to the WT case, where fewer H-bonds are established and mainly across monomers.

### Removal of residues 1–29 in degraded EcAII reveals a new predicted epitope

*In silico* epitope prediction allows to highlight the presence of protein sequences able to induce an immune response *in vivo* according to bioinformatic algorithms. Degradation of the wild-type implies removal of the N-terminal loop, and exposure of an otherwise hidden patch of protein surface. The increased stability of the N24S mutant can prevent this exposure and the generation of antibodies directed towards this surface patch. Indeed, prediction on degraded EcAII (i.e. missing residues 1–29 or 1–24) by both Ellipro and DiscoTope allowed to highlight a novel epitope, present at the intimate dimer interface and involving residues 117–120, which is not predicted in the intact WT protein.

## Discussion

ASNase-based therapy has an outstanding efficacy in paediatric ALL treatment^25^, but roughly 10% of treated patients are not responsive to the standard combination therapy and 10–20% of children in remission experience relapse within 5 years. Metabolic distress induced by L-Asn and L-Gln depletion, host immune response and ASNase limited stability *in vivo* are among the most important causes of therapy failure^26^. In fact, early development of anti-ASNase antibodies is linked to allergic reactions and negative outcome even in the absence of allergic symptoms^25,27^. Beyond native immunogenic epitopes, tumour-released proteases can degrade ASNase, unmasking new sites and increasing the risk of antibody production^6^. Because Asn24 is the primary EcAII CTSB and AEP cutting site^6,15^, we designed an N24S mutant to test if a shorter side chain preserving an oxygen group could lead to protease resistance and improved *in vitro* efficacy. Though enzymatically equivalent to the WT, N24S showed higher stability upon medium and long-term storage and the capacity of maintaining its functional folding in harsh thermal conditions. Investigation of the structural basis of these unique features evidenced that, as expected from the N24S catalytic properties, the enzyme active site was preserved in its architecture (Supplementary Figure 1). The shortening of the Ser24 side chain, however, was responsible for significant changes in the local bond network. These results are in accordance with the ones obtained by thermal shift analysis, reinforcing the hypothesis that the mutant improved stability is mainly linked to the active site loop structural stabilization. This moving structural element includes residues 3–27 and, beyond the mutated Asn24, essential residues for catalysis. Indeed, N24S structure motion for the active site flexible loop is less prominent than the WT one, especially in the region (amino acids 23–26) neighbouring the mutated residue. This increased loop rigidity could also contribute to the enhanced long-term stability of the protein and to a reduced immunogenicity. In fact, the loop seems to protect an interface epitope which could become accessible to the host immune system upon protein degradation.

The strict relationship between loop structural integrity and protein functionality is, in fact, confirmed by the observation that the spontaneous loss of enzyme activity is not related to random degradation, but to removal of the first 25–30 residues carrying the flexible loop. Sample contamination by proteases could not be considered the cause for several reasons: (i) there are no known proteases able to cut this kind of peptide in *E*. *coli* ^28^; (ii) even though N24S and EcAII are prepared in similar ways, spontaneous degradation is consistently present only in the latter; (iii) generation of the 3 cleavage products detected by N-terminal sequencing (starting at amino acid Tyr25, Lys29 and Val30) would require three specific proteases. In this respect, simulation of protease cleavage by Expasy shows that the proteases able to cleave at 25 and 29 are not present in *E*. *coli* ^28^ (Supplementary Material), but in Mammals; (iv) the absence of proteases in the protein sample was definitely demonstrated by incubation with casein.

Spontaneous cleavage of the peptide bond next to asparagine or aspartate in the presence of water is, in fact, a common undesirable reaction in stored proteins^29^. The pathway of this cleavage reaction is closely related to the one leading to deamidation of asparagine side chains and the isomerization of aspartate residues. This requires cyclization involving the side-chain carbonyl carbon of asparagine and the next amide NH group, resulting in a succinimide derivative that then hydrolizes to aspartic acid derivatives. Normally, this occurs through pre-equilibrium deprotonation of the NH group next to the Asn or Asp residue. In case the latter is not available (for example in prolines or in the presence of bulky side chains), pre-equilibrium deprotonation of the amide group of asparagine is followed by a nucleophilic attack by the nitrogen atom of the amide anion on the peptide carbonyl carbon of the asparagine residue. This gives peptide chain cleavage.

In our case, the wild type protein indeed carries an Asn in the solvent exposed position 24, which is replaced by a Ser in the N24S mutant. Moreovoer, residue 25 is a Tyr, perfectly emboding the situation described above leading to peptide cleavage.

N24S exerts a 50% enhanced cytotoxicity compared to the wild-type in long-term exposure to protease secreting SD1 leukaemia cells. Its outstanding resistance to protease cleavage compared to WT is at the core of the successful performance on this cell line, representative of high-risk leukaemia clones. Protease secretion, particularly of AEP and CTSB^7^, is a known feature of Ph+ and iAMP21 ALL, both characterized by a poor outcome^8,30^. These two leukaemia forms account for 4–6% of total ALL cases and their therapy is at the moment an unmet need. For its features, N24S represents an ideal candidate for a new therapeutic strategy. Its resistance to AEP cleavage at sites C-terminal to an Asn residue derives from Asn24 removal. The latter also reduces the cooperative effect of the NY sequence pair (residues 24–25) normally favouring CTSB cleavage^31^.

At least three mechanisms seem therefore to be at the basis of N24S increased long-term stability: an increased chemical resistance to hydrolysis, a stabilization of the active site loop bond network, and a general rigidification of the monomer structure. The sequence modification is also responsible for increased protease resistance.

Improved protein stability has *per se* great potential for improving ALL therapy. N24S EcAII could be easily combined with innovative drug delivery technologies like GRASPA, which successfully reduces ASNase immunogenicity and improves its half-life^32,33^, or chemical modifications like PEGylation, which, in PEGaspase (Oncaspar), accounts for its improved stability *in vivo* and for its highly reduced immunogenicity^34^. Interestingly, Oncaspar is nevertheless still sensitive to CTSB degradation^6^ and, therefore, not ideal for the treatment of CTSB-overexpressing leukaemias.

The improved features of N24S offer a wide spectrum of new uses for the drug. For example, ASNases efficacy in solid tumours has been shown to be discontinuous and highly tumour dependent^35–37^. Increased protease activity, including CTSB’s, in solid tumours, derives not only from the tumour mass, but also from the cells surrounding the tumour, with relevance in carcinogenesis and tumour metastasis^38^. ASNase *in vitro* activity has been reported against gastrointestinal, lung and breast cancers, and multiple myeloma^39–41^. Phase I and phase I-II clinical trials using PEGaspase were also conducted in adult patients with solid tumors^40,42^. However, a large portion of the treated patients was not responsive to the treatment mainly because the active dose of the drug quickly decreased after a few administrations^40,42^. Proteolytic inactivation of the drug could be a valid explanation of its low efficacy. Additional studies on spontaneous and/or protease-mediated degradation of ASNase in cell culture media, animal and patient sera are therefore needed to evaluate the practicality of a protease resistant version of the enzyme in the treatment of solid tumours.

According to our observations, though *in vivo* data will be essential, N24S is potentially useful to counteract limitations (ii)-(iv) mentioned in the Introduction because of its increased stability, conditioning: (i) a reduction of immunogenicity due to the removal of one predicted epitope; (ii) a higher *in vivo* efficacy inferred from the improved *in vitro* one; (iii) a higher *in vivo* stability, as one mechanism of decay, beyond the antibody response, derives from the wild-type sensitivity to proteases.

In summary, our results show that N24S presents outstanding features for an improved ASNase-therapy: preserved functional parameters, improved long-term stability in culture and upon storage, preserved or improved IC_50_, and resistance to tumour-derived proteases. It appears therefore as a potentially suitable alternative to the present routinely used formulations to reduce administration frequency, but especially for the treatment of refractory, relapsing and adult patients.

Moreover, protease resistance of N24S could be exploited for the treatment of solid tumours over-expressing CTSB that have thus far shown little or absent *in vivo* sensitivity to ASNase-based treatments. Further *in vivo* studies will hopefully confirm the improved features of N24S and pave the way for a safer, more effective ASNase-based therapy in the clinics.

## Materials and Methods

### Cloning

The construct containing the *Escherichia coli ansB* gene (Reference sequence: NC_000913.2^43^), devoid of the leader sequence (nt. 1–63) and with a 5’ sequence encoding a 6xHisTag, was amplified from a synthetic version of the full-length gene (pMA EcAnsB, GeneArt). The obtained amplification product was used for cloning into the pET101/D-TOPO (Invitrogen) expression vector. The final construct was used as a template for site-directed mutagenesis.

The pET101/D-TOPO vector containing the WT or the N24S mutated *ansB* gene was used to transform *Escherichia coli* BL21(DE3) *ΔansA/ΔansB* cells, a BL21(DE3) derivative strain designed to lack *E*. *coli* endogenous *ansA* and *ansB* genes.

### Generation of the BL21(DE3) *ΔansA/ΔansB* strain

Intermediate strains and primer sequences used to generate the BL21(DE3) *ΔansA/ΔansB* strain are listed in Supplementary Tables 3 and 4, respectively. The upstream and downstream flanking regions of the *ansA* coding sequence from *E*. *coli* strain BL21(DE3) (DSM919) were joined together by splicing by overlap extension (SOE) PCR. Primers EC_ansA_SOE_upF_SphI and EC_ansA_SOE_upR were used to amplify a 500 bp region upstream of *ansA* using genomic DNA from BL21(DE3), while primers EC_ansA_SOE_downF and EC_ansA_SOE_downR_XbaI were used to amplify the 500 bp region downstream of *ansA*. The products from the upstream and downstream amplification reactions were mixed and a SOE reaction was performed using the EC_ansA_SOE_upF_SphI and EC_ansA_SOE_downR_XbaI primers. The resulting 1 kb amplicon was subcloned into the pGEMT-Easy vector to generate pDSM1084. Similarly, the upstream and downstream flanking regions of the *ansB* coding sequence from *E*. *coli* strain BL21(DE3) were joined together by SOE PCR. Primers EC_ansB_SOE_upF_SphI and EC_ansB_SOE_upR were used to amplify a 500 bp region upstream of *ansB* using genomic DNA from BL21(DE3), while primers EC_ansB_SOE_downF and EC_ansB_SOE_downR_SphI were used to amplify the 500 bp region downstream of *ansB*. The products from the upstream and downstream amplification reactions were mixed and a SOE reaction was performed using the EC_ansB_SOE_upF_SphI and EC_ansB_SOE_downR_SphI primers. The resulting 1 kb amplicon was subcloned into the pGEMT-Easy vector to generate pDSM1085.

To enable DNA transfer through a DNA donor delivery system, the *ans* gene flanking regions were moved into the pCVD442^44^ vector and then transformed into the Sm10-λpir strain of *E*. *coli*. The pCVD442 vector confers ampicillin resistance and sucrose sensitivity. Briefly, pCVD442 and pDSM1084 were double digested with *Sph* I and *Xba* I, and the 1 kb fragment from pDSM1084 (containing the *ansA* flanking regions) and the pCVD442 backbone were gel purified and ligated. The ligation product was transformed into chemically-competent DH5α-λpir cells. Transformants were selected on LB plates supplemented with 100 µg/ml ampicillin (Amp^100^), and the correct plasmid was verified by restriction digest with *Sph* I and *Xba* I and by amplification with EC_ansA_SOE_upF_SphI and EC_ansA_SOE_downR_XbaI primers. This strain was designated DSM1086. Next, pDSM1086 was isolated from DSM1086 and was transformed into chemically-competent Sm10-λpir cells. Transformants were selected on LB plates supplemented with Amp^100^, and the correct plasmid was verified by restriction digest with *Sph* I and *Xba* I and amplification with EC_ansA_SOE_upF_SphI and EC_ansA_SOE_downR_XbaI primers. The resulting strain was designated as DSM1088. Using a similar strategy, pCVD442 and pDSM1085 were digested with *Sph* I, and the 1 kb fragment from pDSM1085 (containing the *ansB* flanking regions) and the CIP-treated pCVD442 backbone were gel purified and ligated. The ligation product was transformed into chemically-competent DH5α-λpir cells. Transformants were selected on LB plates supplemented with Amp^100^, and the correct plasmid was verified by restriction digest with *Sph* I and amplification with EC_ansB_SOE_upF_SphI and EC_ansB_SOE_downR_SphI primers. This strain was designated DSM1087. Next, pDSM1087 was isolated from DSM1087 and was transformed into chemically-competent Sm10-λpir cells. Transformants were selected on LB plates supplemented with Amp^100^, and the correct plasmid was verified by restriction digest with *Sph* I and amplification with EC_ansB_SOE_upF_SphI and EC_ansB_SOE_downR_SphI primers. The resulting strain was designated at DSM1089.

To develop the *ΔansA* BL21(DE3) strain, strains DSM919 and DSM1088 were grown overnight in LB cultures supplemented with 50 μg/ml streptomycin (Sm^50^) and Amp^100^, respectively. Aliquots from each culture, corrected to obtain the same OD_600_, were pelleted and washed twice with LB. The pellets were resuspended in LB, combined, re-pelleted, and resuspended in LB. The resuspension was placed on an unsupplemented LB plate, and the cells were allowed to mate for 1 hour at 37 °C. The cells were then resuspended in LB and plated on LB agar plates supplemented with Sm^50^ and Amp^100^ to select for BL21(DE3) cells that integrated the pDSM1088 plasmid through a single crossover event. The single colonies were further expanded on LB agar plates supplemented with Sm^50^ and Amp^100^. The streaks were then used to start LB liquid cultures without any antibiotic supplementation. Following overnight growth at 37 °C, the cultures were sub-cultured into LB. Once the sub-cultures were turbid, varying dilutions of the cultures were plated on L-broth plates supplemented with 10% sucrose. Resulting colonies were expanded on 10% sucrose L-broth agar plates and screened using the Ec_ansA_Far_F and Ec_ansA_Far_R primers, which produce a 2 kb band when the *ansA* coding region has been successfully deleted. DSM1092 was identified through this method and the region sequenced using primers Ec_ansA_Farseq_F and Ec_ansA_Farseq_R, which confirmed the deletion of the *ansA* gene. Using the same method described above, strains DSM919 and DSM1089 were mated to develop the *ΔansB* strain, which was screened using the Ec_ansB_Far_F3 and Ec_ansB_Far_R3 primers, producing a 1.6 kb band when the *ansB* coding region has been successfully deleted. The deletion was further confirmed through sequencing with primers Ec_ansB_Farseq_F and Ec_ansB_Farseq_R, and the strain was designated at DSM1093.

To develop the *ΔansA/ΔansB* BL21(DE3) double mutation strain, strains DSM1092 and DSM1089 were grown overnight in LB cultures supplemented with Sm^50^ and Amp^100^, respectively. Aliquots from each culture, corrected to obtain the same OD_600_, were pelleted and washed twice with LB. The pellets were resuspended in LB, combined, re-pelleted, and resuspended in LB. The resuspension was placed on an unsupplemented LB plate, and the cells were allowed to mate for 1 hour at 37 °C. The cells were then resuspended in LB and plated on LB agar plates supplemented with Sm^50^ and Amp^100^ to select for BL21(DE3) *ΔansA* cells that integrated the pDSM1089 plasmid through a single crossover event. The single colonies were further expanded on LB agar plates supplemented with Sm^50^ and Amp^100^. The streaks were then used to start LB cultures without any antibiotic supplementation. Following overnight growth at 37 °C, the cultures were sub-cultured into LB. Once the sub-cultures were turbid, varying dilutions of the cultures were plated on L-broth plates supplemented with 10% sucrose. Colonies were expanded on the 10% sucrose L-broth agar plates and screened using the Ec_ansB_Far_F3 and Ec_ansB_Far_R3 primers, which produce a 1.6 kb band when the *ansB* coding region has been successfully deleted. DSM1096 was identified through this method and sequenced using primers Ec_ansB_Farseq_F and Ec_ansB_Farseq_R to confirm the deletion of the *ansB* gene. Additionally, the deletion of the *ansA* gene was re-confirmed by sequencing using primers Ec_ansA_Farseq_F and Ec_ansA_Farseq_R.

### Protein over-expression and purification

Protein over-expression was obtained according to Studier^45^. Recombinant proteins were purified by a two-steps chromatographic method that consisted in: nickel affinity chromatography and anionic exchange at pH 7.4 (for details, see supplemental Methods). Fractions positive for the enzymatic activity and pure to homogeneity were used for the following experiments. Proteins to be used for cytotoxicity assays were dialyzed *versus* PBS (0.01 M Na/K-phosphate buffer, 0.0027 M KCl and 0.137 M NaCl, pH 7.4). For crystallization, N24S was dialyzed *versus* 30 mM Tris-HCl pH 8.0.

### L-asparaginase and L-glutaminase activity assays

In order to determine ASNase and L-glutaminase activities, two methods were employed: a spectrophotometric one, using the coupled reaction catalyzed by glutamic acid dehydrogenase (GADH), and a colorimetric one, using Nessler’s reagent (for details, see supplemental Methods).

One Unit of L-Asparaginase is defined as the amount of enzyme needed to convert 1 µmole of substrate into the product in the experimental conditions.

### Cell lines

Human Acute Lymphoblastic Leukaemia (ALL) cells employed for the experiments are listed in supplemental Table 3. Human Acute Lymphoblastic Leukaemia cells (MOLT-4, SD1, REH, RS4;11) were grown in RPMI 1640 complete media (10% Fetal Bovine Serum, 2 mM L-glutamine, 50 μg/ml gentamicin). Cells were passaged every other day and cultured in a humidified incubator at 37 °C in the presence of 5% CO_2_.

### Cytotoxicity evaluation

In order to compare the cytotoxicity of the recombinant EcAII WT and N24S proteins to the one of the commercial Elspar enzyme (Medac), 1 × 10^5^ cells were plated in 200 µl complete medium. Elspar was used as an internal control as the commercial protein has the same formulation of the drug used in the clinics and, as opposite to the recombinant EcAIIs, Elspar has no histidine tag. Lyophilized ASNase proteins were resuspended in complete medium and were added to the wells at a final concentration near the IC_50_ value for each cell line^46^. Each experiment was done in triplicate and repeated at least 3 times on different days. Trypan blue exclusion-dye count was done at 72 h and only living cells were counted.

To evaluate the improved stability of N24S lacking the main protease-recognition site^16^, its long-term cytotoxic effect was evaluated. SD1 and REH cell lines were chosen as model systems for the study, as SD1 cells over-express proteases, while REH cells do not. 1 × 10^5^ cells were plated in 1.5 ml complete medium in a 24-well plate. Cells were treated with 0.5 U/ml (SD1) or 1 U/ml (REH). Cell viability was measured by trypan blue living-cell exclusion dye at 72 h and 144 h. Data were expressed as percentage of living cells *vs*. control.

### Protein stability assays

Protein stability was assessed as residual activity (T_50_) and by thermal shift (T_0.5_). To determine the T_50_, the enzyme in Na-phosphate 50 mM pH 7.4 was incubated at different temperatures (range, 37–70 °C) for 10 min. After incubation, the enzyme solution was cooled on ice and the enzyme activity measured by nesslerization. The T_50_ value was determined using the Temperature Melting Plot for Sigma Plot (SPSS Inc.). The treated enzyme residual activity was expressed as the percentage of activity of the heat-treated sample *versus* the untreated one. A thermal shift assay was performed using a MiniOpticon real time system (Bio Rad). Samples (0.5–1.5 mg/ml) in PBS buffer were mixed with 20X SYPRO orange dye (Invitrogen) in a final volume of 50 μl. Samples were centrifuged and heated from 25 to 95 °C at 1 °C or 0.5 °C/min. Fluorescence was monitored at 517 nm. Raw data were normalized so that a value of 1 corresponds to completely unfolded protein. From the obtained curve, the T_0.5_ for each protein was extrapolated.

### Statistical analysis

Long-term cytotoxicity data were analyzed by one-way ANOVA and Tukey test. The normal distribution for each data group was systematically verified by Shapiro-Wilk test^47^ and p values above 0.05 were considered significant for normally distributed data. Each experiment was repeated 3 times and in triplicate for each condition (n = 9); p-values < 0.05 were considered significant. Thermal stability data were analyzed by T-test. Experiments were repeated 3 times for T_50_ determination and 6 times for T_0.5_; p-values < 0.05 were considered significant.

### Western blot analysis

For Western blot analysis, proteins were transferred from within the gel to a PVDF 0.22 µm membrane (Millipore). The blocked membrane was incubated with an HRP-conjugated anti-HisTag antibody (Sigma) diluted 1:7000.

### N-terminal sequencing

In order to determine the N-terminal sequence of partially degraded EcAII WT protein, the Edman method was employed^48^. Briefly, after automated Edman degradation, phenylthiohydantoin(PTH)-derivates were separated by reverse-phase HPLC and the retention time was compared to a previously obtained standard curve of PTH-amino acids. The analysis was done on our behalf at the Laboratory of Proteins Primary Structure Analysis - “Centro grandi strumenti”, University of Pavia. For the analysis, partially degraded protein solution pure to homogeneity was provided at a concentration of 6.8 nM in PBS buffer. The solution was directly loaded onto a biphasic column connected to an automatic sequencer (HP G1000A, Agilent). After 12 degradation cycles, data were analyzed.

### Intact protein Mass Spectrometry

Samples previously prepared for SDS-PAGE were precipitated with chloroform/methanol and analyzed by liquid chromatography-mass spectrometry (LC-MS), as described previously^49^. Briefly, water was added to a final volume of 100 µL followed by 300 µL methanol and then 100 µL chloroform with mixing. An additional 200 µL water was then added to induce phase separation and the sample vortex mixed for 1 min. The protein precipitate was brought to the interface by centrifugation at 15,000 × g for 2 min. The upper phase was discarded and 300 µL methanol added prior to mixing the single organic phase. The protein precipitate was recovered by centrifugation at 15,000 × g for 2 min, the supernatant removed and the pellet dried at room temperature and pressure for 5 min. The pellet was dissolved in 70% formic acid and immediately injected onto an equilibrated reverse-phase column (Agilent PLRP/S, 150 × 2 mm, 300 Å, 5 µm, 40 °C). The column was equilibrated in 95% buffer A (0.1% TFA in water), 5% buffer B (0.05% TFA, 50% isopropanol, 50% acetonitrile) at 200 µL/min for 20 min prior to sample injection and initiation of a linear gradient, 5% B at 5 min, 90% B at 55 min. The column eluent was directed to an electrospray-ionization source (Ionmax, Thermo) on a linear ion-trap mass spectrometer operated in positive ion mode (LTQ, Thermo) and scanned from 600–2000 m/z. The asparaginase peak was the only discernable peak in the LC-MS run and was deconvoluted using Magtran software. Mass accuracy on the LTQ typically gives better than 0.02% error on protein molecular weight determinations.

### Protease sensitivity test

Confluent SD1 cells were collected by centrifugation (3500 rpm, 4 °C, 5 min) and washed twice with PBS. The washed cell pellet was resuspended in lysis buffer (50 mM trisodium acetate buffer, pH 4.5, 5 mM DTT) using a 1 × 10^6^ cells:0.1 ml buffer ratio. Cells were lysed by 4 cycles of freeze-thawing at −80 °C. Cell lysate was clarified by centrifugation (8000 rpm, 10 min, 4 °C) and whole protein extract was quantified by bicinchoninic acid assay (BCA, Pierce). The final reaction mixture volume was 50 μl and contained: 100 μg cell lysate (only for treated samples) and 4 μg ASNase. After 24 h incubation at 37 °C protein separation in denaturing and reducing conditions was performed by electrophoresis using a precast gradient 4–12% polyacrylamide gel (Bolt^®^ gel, ThermoFisher Scientific). Proteins were transferred onto a nitrocellulose membrane using an iBlot^®^ 2 device (ThermoFisher Scientific). The membrane was stained using Ponceau Red dye. Beyond EcAII wild type and N24S, also commercial Elspar was tested for proteases sensitivity as it was already used in previous similar experiments^6,50^ and, mainly, as it has the same formulation of the drug used in the clinics.

### Crystallization

*Escherichia coli* type II ASNase N24S mutant was purified as reported above. The pure protein fraction was dialyzed versus 30 mM Tris-HCl, pH 8.0 and used for crystallization. Crystallization attempts were carried out using a sparse-matrix screening from Molecular Dimensions (MD1-03). The crystals were grown at 21 °C using the sitting-drop vapour diffusion method and by mixing equal volumes of mother liquor and 4 mg/ml protein solution in a 2 µl drop. The original crystallization condition contained 100 mM HEPES, pH 7.5, 10% w/v PEG 8000, 8% v/v ethylene glycol. In order to improve crystal quality the condition was optimized varying buffer pH and precipitants concentration. Upon cryo-mounting on nylon loops, crystals to be analyzed by X-ray were soaked into 100 μM L-aspartic acid (L-Asp) dissolved in mother liquor for 10 min.

### Structure solution

X-ray data were collected at 100 K, 0.98 Å at beamline ID-29 at the European Synchrotron Research Facility (ESRF), Grenoble, France^51^. Data were integrated with Mosflm^52^ and merged with SCALA^53^. The space group was determined with POINTLESS^53^. The structure was solved by molecular-replacement with PHENIX-MR^54^ and using an intimate dimer of *E*. *coli* WT asparaginase (PDB ID: 3ECA)^55^ as a search model. Structure refinement was done with PHENIX-REFINE^56^ starting from an initial model deprived of ligand and of residues of the active-site flexible loop (residues 8–35). At each refinement run, residues were added manually where clear electron density was present. Ligands were added with PHENIX-LIGAND FIT and using L-aspartic acid as a search model^54^. Ramachandran statistics after refinement were the following: 98.58% lay in the favoured region, 1.42% in the allowed region and no outliers were present.

### Normal Mode-based geometric Simulations

The crystallographic structures of monomer A from WT (1NNS) and monomer B from N24S (5MQ5) in complex with the L-aspartate product, and hence in the more compact conformation, were used for Normal Mode-based geometric simulations (NMSim)^57^. Loop motion was analyzed by small scale motions, whole protein motions were analyzed both with the lowest and largest radius of gyration (ROG) using default parameters (http://cpclab.uni-duesseldorf.de/nmsim/main.php). 0.5 Å was chosen as the ΔÅ cut off for relevance considering the structures high atomic resolution (1.95 Å WT and 1.60 Å N24S), the elevated number of simulation cycles (e.g., 500) and the average Δ (0.46 Å).

### Molecular Dynamics simulations

WT and N24S L-Asparaginase were studied with all atom Molecular Dynamics (MD) simulations in the apo state and bound to Aspartate. Simulations of the bound forms were started from the respective crystal structures of WT (PDB ID: 3ECA) and N24S (PDB ID: 5MQ5) while starting structures for the apo forms were generated by simply deleting the coordinates of the ligand from the respective crystals.

We performed energy minimization of all the starting structures with AMBER16^58^ in explicit solvent (TIP3P water model^59^) using the FF14SB force field^60^ with a steepest descent method (3 × 10^3^ steps), followed by a run in conjugate gradient algorithm (7 × 10^3^ steps). After minimization, we heated the system from 0 to 300 K in the NVT ensemble over 25000 MD steps with a timestep of 2 fs. After the heating process, the simulation was run for 50 ps (2 fs timestep) in the NPT ensemble (Berendsen barostat, Langevin temperature control with γ = 2 ps^−1^) using the SHAKE algorithm to constrain all the hydrogen-containing bonds^61^.

All production simulations were performed in the same condition of the last equilibration part; we carried out MD simulations of the crystal structures of WT and N24S ASNase at 300 K (100 ns each). Calculations of Root Mean Square Deviation (RMSD) and Root Mean Square Fluctations (RMSF) were performed using the tools available in the AMBER suite.

### Protein internal dynamics

In order to analyze the impact of the N24S mutation on the internal dynamics properties of ASNase that can be related to global structural stabilization, we made use of the previously introduced distance fluctuation analysis. For each MD trajectory in different bound states, we computed the matrix of distance fluctuations, in which each element of the matrix corresponds to the DF parameter:

$${{\rm{DF}}}_{{\rm{ij}}}=\langle {({{\rm{d}}}_{{\rm{ij}}}-\langle {{\rm{d}}}_{{\rm{ij}}}\rangle \rangle }^{2}\rangle $$

where d_ij_ is the (time-dependent) distance of the Cα atoms of amino acids i and j and the brackets indicate the time-average over the trajectory. This parameter is invariant under translations and rotations of the molecules and, unlike the covariance matrix, does not depend on the choice of a particular protein reference structure. The DF matrix can be used to assess the intrinsic flexibility/rigidity of the proteins, and how these properties change upon mutation.

The DF was calculated for any pair of residues during the trajectory. This parameter characterizes residues that move in a highly coordinated fashion, and it is actually able to reflect the presence of specific coordination patterns. Proteins that are highly coordinated may show enhanced stability^22,62,63^.

### Epitope prediction

The tetrameric EcAII WT structure (PDB: 3ECA), either full-length or missing residues 1–24 or 1–29, was used for epitope prediction using the online, open source software ElliPro^64^ and Discotope^65^. Protein truncation at amino acid 24 was chosen to simulate CTSB and AEP cleavage; truncation at amino acid 29 was instead chosen according to N-terminal sequencing results for degraded EcAII. Default parameters and a score threshold equal to 0.5 were used to identify relevant epitopes. A similar analysis was performed on the N24S mutant structure (PDB: 5MQ5).

### Equipment and settings

For Fig. 2, all images were acquired using an EPSON DX4050 scanner at 300 dpi. Panels were assembled using Microsoft Power point. The blot image reported in panel d was cropped and brightness was adjusted across the whole image (full-length, unmodified blot is presented in Supplementary Figure 3). For Fig. 4, the image was also acquired using an EPSON DX4050 scanner at 300 dpi. The image mode was changed from RGB color to Grayscale using Adobe Photoshop 6.0. Full-length, unmodified blot is presented in Supplementary Figure 4.

### Data Availability

The N24S structure is available in the Protein Structure Database under ID 5MQ5. N24S is covered by patent filing number PCT/EP2016/076994.

## Electronic supplementary material

Supplementary data
