# Supplementary material for: A protease-resistant Escherichia coli asparaginase with outstanding stability and enhanced anti-leukaemic activity in vitro
Source: Sci Rep. 2017 Nov 3;7:14479. doi: 10.1038/s41598-017-15075-4 (PMC5670125; doi:10.1038/s41598-017-15075-4)
Supplement: Supplementary file 1 — Supplementary data [file 41598_2017_15075_MOESM1_ESM.doc]

**A protease-resistant *Escherichia coli* asparaginase with outstanding stability and enhanced anti-leukemic activity *in vitro***

Maristella Maggi, Steven D. Mittelman, Jean Hugues Parmentier, Giorgio Colombo, Massimiliano Meli, Jeannette Marie Whitmire, Douglas Scott Merrell, Julian Whitelegge, Claudia Scotti

**Data supplement**

**Materials and Methods**

**Protein purification**

Cell extract was applied to a HisTrap 5 ml column equilibrated in buffer NA (50 mM Na-phosphate, 300 mM NaCl, 10 mM imidazole pH 8.0) at 2.0 ml/min. Proteins were eluted using a step gradient (25, 50, 100, 250, 500 mM imidazole) and 5 ml fractions were collected.

Fractions were checked for ASNase activity and analyzed by SDS-PAGE using a 12% acrylamide/bis-acrylamide gel. The eluted protein sample was applied to a 1 ml HiTrap Q XL column equilibrated in buffer QA (50 mM Na-phosphate pH 7.4). The flow-through fraction was collected and the bound proteins were eluted using elution buffer with a high salt concentration (50 mM Na-phosphate, 1M NaCl pH 7.4). Both the flow-through and the bound fractions were checked for ASNase activity. An aliquot was analyzed by SDS-PAGE using a 12% acrylamide/bis-acrylamide gel.

**L-asparaginase and L-glutaminase activity assays**

The standard reaction mixture used to measure ammonia release contained: 50 mM 4-(2-Hydroxyethyl)piperazine-1-ethanesulfonic acid (HEPES) buffer pH 7.5, 1 mM α-ketoglutarate (α-KG), 0.24 mM NADH, 20 U glutamic acid dehydrogenase and 10 mM L-ASN or 10 mM L-GLN ^1^, in a final volume of 0.5 ml. The reaction was started by adding enzyme solution (5-300 μg) and NADH consumption was monitored at 340 nm with a single-ray spectrophotometer. The standard protocol for ammonia determination using Nessler's reagent was adjusted for analysis in a 96-well plate. Briefly, a solution containing 50 mM Hepes pH 7.5, 0-10 mM L-ASN or 0-10 mM L-GLN and 5-30 µg enzyme solution was incubated at 37°C for 15 min for L-asparaginase activity determination or for 30 min for L-glutaminase activity. The reaction was then stopped adding 0.075 M tricloracetic acid (TCA) on ice for 15 min. After centrifugation (14000 x g, 15 min), 10 µl reaction were used for the analysis with Nessler's reagent. According to the manufacturer instructions, Nessler's reagent was diluted 10-fold and 90 µl of working solution were added to 10 µl reaction in a final volume of 100 µl. In all cases, a negative control without enzyme was set up in order to determine the background noise due to spontaneous release of ammonia from the substrates. k_cat_ or turn-over number is the number of catalytic events per second. K_m_ is the substrate concentration at which the reaction speed is half-maximal. The kinetic parameter were determined by Lineweaver-Burk plot using the Enzyme Kinetic Module 1.1 for Sigma Plot (SPSS Inc.).

k_cat_/K_m_ is an index of catalytic efficiency at sub-saturating substrate concentration.

**Cell lines**

Human leukaemia cell lines were obtained from the ATCC (U.S.A.). Each cell line was selected as representative of peculiar ALL aspects (e.g., L-asparaginases sensitivity, ASN-synthetase expression levels, addiction to L-Asn or to L-Gln, L-asparaginases inactivating proteases expression). Particularly: (i) MOLT-4 are T-lymphoblasts derived from a relapsing 19-years old patient, the cells showed high sensitivity to L-asparaginases, low to absent expression of ASN-synthetase and moderate addiction to L-Asn and to L-Gln; (ii) RS4;11 are lymphoblasts derived from a 32-years old ALL patient, the cell line has high sensitivity to L-asparaginases, absent ASN-synthetase expression and high addiction to L-Asn; (iii) SD1 cell line is a lymphoblasts model cell line carrying the chromosomal mutation t(9;22) Ph1 BCR/ABL that often correlates with highly aggressive and poorly responsive ALL both in children and adult, the cell line is reported to over-express L-asparaginases inactivating proteases, to have low to moderate ASN-synthetase expression levels and high addiction to L-Gln; (iv) REH cells are non-T, non-B lymphoblasts derived from an ALL patient, the cell line is discontinuously sensitive to L-asparaginases and does not over-express L-asparaginases inactivating proteases.

**Tables**

**Supplementary Table 1** List and description of *Escherichia coli* strains used to generate the BL21(DE3) *ΔansA/ΔansB* strain.

| **Strain name** | **Description** |
| --- | --- |
| DE3 | *Escherichia coli* BL21(DE3) |
| DH5α-λpir | *E. coli* DH5α-λpir |
| Sm10-λpir | *E. coli* Sm10-λpir |
| DSM919 | *E. coli* BL21(DE3), Sm^R^ |
| DSM1084 | *E. coli* Top10 (pGEM T-easy:: *ansA*-flank), Amp^R^ |
| DSM1085 | *E. coli* Top10 (pGEM T-easy:: *ansB*-flank), Amp^R^ |
| DSM1086 | *E. coli* DH5α-λpir (pCVD442:*ansA*-flank), Amp^R^, Suc^S^ |
| DSM1087 | *E. coli* DH5α-λpir (pCVD442:*ansB*-flank), Amp^R^, Suc^S^ |
| DSM1088 | *E. coli* Sm10-λpir (pCVD442:*ansA*-flank), Amp^R^, Suc^S^ |
| DSM1089 | *E. coli* Sm10-λpir (pCVD442:*ansB*-flank), Amp^R^, Suc^S^ |
| DSM1092 | *E. coli* BL21(DE3) (*ΔansA*), Sm^R^ |
| DSM1093 | *E. coli* BL21(DE3) (*ΔansB*), Sm^R^ |
| DSM1096 | *E. coli* BL21(DE3) (*ΔansA/ΔansB*), Sm^R^ |

**Supplementary Table 2** List and sequences of primers used to generate the BL21(DE3) *ΔansA/ΔansB* strain.

| **Name** | **Sequence** |
| --- | --- |
| EC_ansA_SOE_upF_SphI | ACATGCATGCGGGCCACGTCTGGACCGGTC |
| EC_ansA_SOE_upR | GAGGGGGCATTACAGTCTCCGATATTGATATGAATGATATCGAAAGAGC |
| EC_ansA_SOE_downF | GCTCTTTCGATATCATTCATATCAATATCGGAGACTGTAATGCCCCCTC |
| EC_ansA_SOE_downR_XbaI | TGCTCTAGACGTCCAGCACGGTAAACTTCACG |
| EC_ansB_SOE_upR | CGTTCACGTAACTGGAGGAATGAATCACTTCGCCCCGGTATCGTGC |
| EC_ansB_SOE_downF | GCACGATACCGGGGCGAAGTGATTCATTCCTCCAGTTACGTGAACG |
| EC_ansB_SOE_downR_SphI | ACATGCATGCCCGCATTATCGAAACGCGCAG |
| Ec_ansA_Far_F | GAAACTCAGGGGAATGTTGG |
| Ec_ansA_Far_R | CTTCGCGTCGGCGGTTTATATC |
| Ec_ansA_Farseq_F | CATTCGACGCCGGAGCAGATTG |
| Ec_ansA_Farseq_R | CACGCCACGACAACCATCG |
| Ec_ansB_Far_F3 | GCTGAATCGGCACTTCATAACAC |
| Ec_ansB_Far_R3 | GCACTTTCAGTGACGGCAATG |
| Ec_ansB_Farseq_F | GAAATCTTCGCTATTCACGATCAG |
| Ec_ansB_Farseq_R | CGTCTGACCAAACTTGATGCGC |
| EC_ansB_SOE_upR | CGTTCACGTAACTGGAGGAATGAATCACTTCGCCCCGGTATCGTGC |

**Supplementary table 3** Human ALL cell lines features.

| **Cell line** | **Cell type** | **Culture properties** | **Genomic**  **characteristics** | **L-ASN or**  **L-GLN addiction^a^** | **L-ASNS**  **expression^a^** |
| --- | --- | --- | --- | --- | --- |
| **MOLT-4** | T-lymphoblast | Suspension,  forming clumps | Hypertetraploid | Moderate | Low to absent |
| **RS4;11** | Pro-B lymphoblast | Suspension | t(4;11)(q21;q23) | High for  L-ASN | Absent |
| **SD1** | Pre-B lymphoblast | Suspension,  forming clumps | Tetraploid, t(9;22) Ph1 BCR/ABL | High for  L-GLN | Low to moderate |
| **REH** | Pro-B lymphoblast | Suspension,  forming clumps | t(12;21)  TEL/AML1 | ND | ND |
| ^a 2^; ND: not determined | | | | | |

**Supplementary Table 4** R.m.s. main chain deviations of 5MQ5 (N24S), 1NNS (WT) and 3ECA (WT) monomers.

| **R.m.s.d. (Å)** | **5MQ5 second chain** | | | | **1NNS chain** | | **3ECA chain** | | | |
| --- | --- | --- | --- | --- | --- | --- | --- | --- | --- | --- |
| **5MQ5 first chain** | **A** | **C** | **B** | **D** | **A** | **B** | **A** | **B** | **C** | **D** |
| **A** | 0.0 | 0.19 | 0.23 | 0.13 | 0.31 | 0.31 | 0.32 | 0.30 | 0.35 | 0.33 |
| C | 0.19 | 0.0 | 0.13 | 0.22 | 0.30 | 0.29 | 0.32 | 0.38 | 0.47 | 0.38 |
| B | 0.23 | 0.13 | 0.0 | 0.23 | 0.32 | 0.30 | 0.32 | 0.40 | 0.49 | 0.39 |
| D | 0.13 | 0.22 | 0.23 | 0.0 | 0.32 | 0.33 | 0.34 | 0.33 | 0.36 | 0.36 |

**Supplementary Table 5** R.m.s. deviations of 5MQ5 (N24S) and 1NNS (WT).

| **Residues selection** | **Main chain r.m.s.d. (Å)** | **All atoms r.m.s.d. (Å)** |
| --- | --- | --- |
| 1 - 326 | 0.19 ± 0.05 | 0.54 ± 0.08 |
| 1 - 37 | 0.22 ± 0.04 | 0.83 ± 0.06 |
| 14 - 27 | 0.17 ± 0.00 | 0.68 ± 0.00 |
| 26 - 46 | 0.21 ± 0.04 | 1.17 ± 0.25 |

**Supplementary Table 6** Proteases able to cleave in position 25 and 29 (EcAII wild type) obtained from simulation of protease cleavage by Expasy PeptideCutter tool^3^.

| **Position of cleavage site** | **Name of cleaving enzyme(s)** | **Resulting peptide sequence** | **Peptide length [aa]** |
| --- | --- | --- | --- |
| 25 | Chymotrypsin-high specificity  Chymotrypsin-low specificity  Pepsin (pH>2) Proteinase K | LPNITILATGGTIAGGGDSATKSNY | 25 |
| 29 | LysC Thermolysin Trypsin | LPNITILATGGTIAGGGDSATKSNY TVGK | 29 |

**
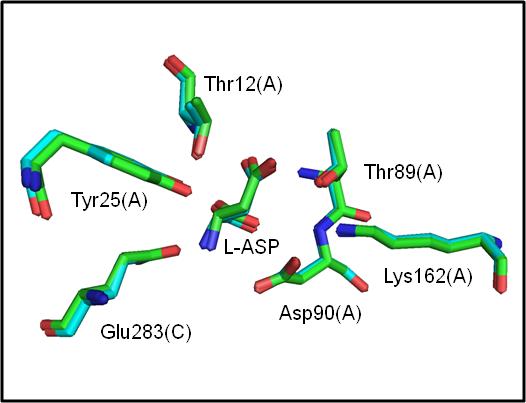
**

**Supplementary Figure 1** EcAII wild type (green) and N24S (cyan) catalytic residues superimposition. For each residue the monomer to which it belongs is reported in capital letters in parentheses. The L-ASP reaction product is reported as well and represented as sticks colored like the structures it belongs to.

**

**

**Supplementary Figure 2. Geometric transition simulations r.m.s.f. and r.m.s.d. Δ values.** (a) Single residue (1-326) small motion. (b) Smallest ROG whole protein motion. (c) Largest ROG whole protein motion. (d) Single residue small motion with largest ROG. Data were calculated by subtracting the motion values obtained for the WT protein from the values obtained for the N24S mutant, therefore negative values indicate reduced motion for single residues (panels a and d) or whole protein (panels b and c) in the mutant compared to the WT.


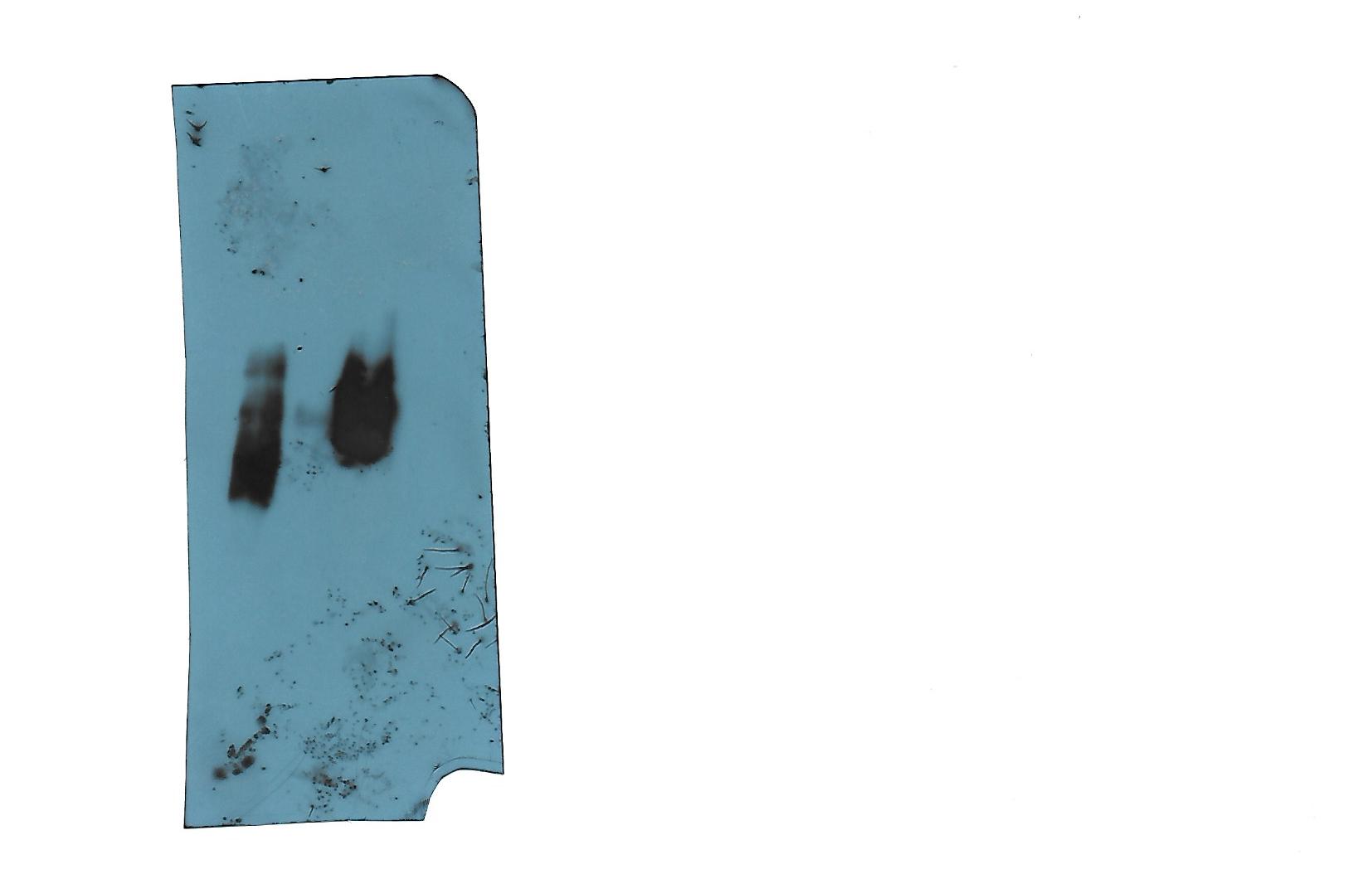


**Supplementary Figure 3**. Original Western blot film wherefrom manuscript Figure 2 was derived.


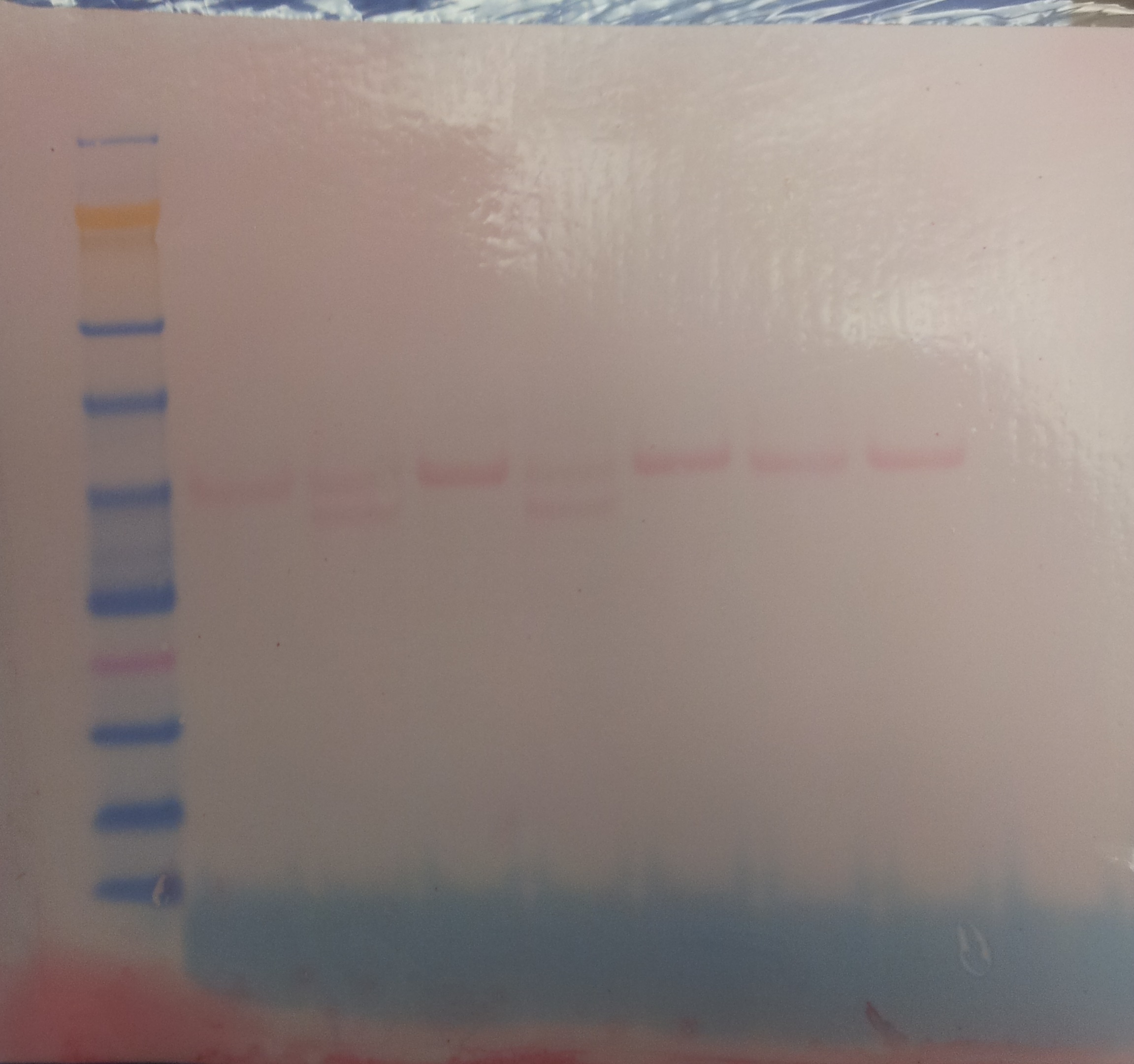


**Supplementary Figure 4.** Original membrane wherefrom manuscript Figure 4 was derived.

**References**

1 Balcão, V. M., Mateo, C., Fernández-Lafuente, R., Malcata, F. X. & Guisán, J. M. Coimmobilization of L-asparaginase and glutamate dehydrogenase onto highly activated supports. *Enzyme Microb Technol* **28**, 696-704 (2001).

2 Ehsanipour, E. A. *et al.* Adipocytes cause leukemia cell resistance to L-asparaginase via release of glutamine. *Cancer Res* **73**, 2998-3006, doi:10.1158/0008-5472.CAN-12-4402 (2013).

3 Wilkins, M. R. *et al.* Protein identification and analysis tools in the ExPASy server. *Methods Mol Biol* **112**, 531-552 (1999).
